# Complete Genome Sequence of *Klebsiella pneumoniae* Sequence Type 17, a Multidrug-Resistant Strain Isolated during Tigecycline Treatment

**DOI:** 10.1128/genomeA.01337-14

**Published:** 2014-12-24

**Authors:** Xiaoting Hua, Qiong Chen, Xi Li, Ye Feng, Zhi Ruan, Yunsong Yu

**Affiliations:** a Department of Infectious Diseases, Sir Run Run Shaw Hospital, College of Medicine, Zhejiang University, Hangzhou, Zhejiang, People’s Republic of China; b Institute for Translational Medicine, Zhejiang University School of Medicine, Hangzhou, Zhejiang, People’s Republic of China; c Clinical Laboratory, Sir Run Run Shaw Hospital, College of Medicine, Zhejiang University, Hangzhou, Zhejiang, People’s Republic of China

## Abstract

*Klbesiella pneumoniae* is one of the most important human pathogens and frequently causes many diseases. To facilitate the comparative genome analysis in tigecycline resistance mechanism, we report the complete chromosomal sequence of a multidrug-resistance *K. pneumoniae* strain before tigecycline treatment for reference genome.

## GENOME ANNOUNCEMENT

*Klbesiella pneumoniae* is a Gram-negative rod-shaped bacteria belonging to the *Enterobacteriaceae* family (1). It is considered one of the most important human pathogens and frequently causes many diseases, including pneumonia, urinary tract infection, and septicemia (2, 3). The rise of multidrug-resistance in *K. pneumoniae* has raised serious therapeutic challenges (4) because patients infected by carbapenem-resistant KPC-producing *K. pneumoniae* have shown high rates of mortality (5). *K. pneumoniae* acquired multidrug resistance through horizontal gene transfer via mobile genetic elements, including carbapenemases (6). That might explain why it spreads quickly in the hospital environment.

XH209 is a multidrug-resistant *K. pneumoniae* strain isolated from the blood of a patient in Hangzhou, Zhejiang, China, during tigecycline treatment. It could be a reference genome for further comparative analysis of tigecycline resistance in *K. pneumoniae*. The strain belongs to sequence type (ST) 17. The strain demonstrated multiple resistances to clinically used antibiotics, including all β-lactams, sulfonamides, and tetracycline, but it was susceptible to tigecycline and colistin.

A single colony of *K. pneumoniae* XH209 was grown in 2 mL of Müller-Hinton broth overnight at 37°C. The genomic DNA of *K. pneumoniae* was extracted using the QIAamp DNA minikit (Qiagen, Valencia, CA, USA) following the manufacturer’s protocol (7). The quality and quantity of extracted genomic DNA were determined by agarose gel and NanoDrop spectrophotometer. Five µg of genomic DNA was used to construct a 300-bp library for Illumina paired-end sequencing. Furthermore, to finish the complete genome of XH209, an 8-kb mate-pair library was also prepared for XH209. The raw Illumina sequencing data was *de novo* assembled via IDBA-Hybrid (8). Then, SSPACE was used to scaffold the preassembled contigs (9), and GapFiller was used to close the gaps within the scaffolds (10). The rest of the gaps were filled by PCR and Sanger sequencing.

The complete genome of *K. pneumoniae* strain XH209 comprised 5,118,878 bp with a GC content of 57.63%. Automatic genome annotation was performed using the NCBI Prokaryotic Genome Annotation Pipeline (http://www.ncbi.nlm.nih.gov/genome/annotation_prok), which predicted a total of 5,023 genes, 4,881 coding sequences, 28 pseudogenes, 25 rRNAs (5S, 16S, 23S), 85 tRNAs, 4 noncoding RNAs, and 20 frameshifted genes.

As mentioned previously, *K. pneumoniae* XH209 was isolated during tigecycline treatment. Furthermore, six independent laboratory evolution mutants were obtained after treating strain XH209 with an increased concentration of tigecycline. Whole-genome sequencing and transcriptome experiment were performed to investigate the tigecycline-resistance mechanism in *K. pneumoniae* and will be reported in a future publication*.*

### Nucleotide sequence accession number.

This complete genome sequence of *K. pneumoniae* XH209 has been deposited at DDBJ/EMBL/GenBank under the accession number CP009461.
